# SYMPTOM BURDEN AND HEALTH CARE UTILIZATION AMONG OLDER ADULTS LIVING WITH MULTIMORBIDITY: THE COORDINATE PROGRAM

**DOI:** 10.1093/geroni/igae098.3023

**Published:** 2024-12-31

**Authors:** Chitchanok Benjasirisan, Arum Lim, Jordan Tebay, Cheryl Dennison Himmelfarb, Patricia Davidson, Binu Koirala

**Affiliations:** Johns Hopkins University, Baltimore, Maryland, United States; Johns Hopkins University, Baltimore, Maryland, United States; Johns Hopkins University, Baltimore, Maryland, United States; Johns Hopkins University, Baltimore, Maryland, United States; University of Wollongong, Wollongong, New South Wales, Australia; Johns Hopkins University, Baltimore, Maryland, United States

## Abstract

The increasing challenges of multimorbidity in older adults underscore the need for sustainable approaches to address care coordination and symptom management. The Care cOORDInatioN And sympTom managEment (COORDINATE) program was developed using experience-based co-design and human-centered principles by considering the needs of patients, family caregivers, and healthcare providers. The program included a need assessment, question prompt list, goals discussion, and symptom assessment in 5 visits over 6-week periods employed among adults aged 55 years and older who were living with at least 2 chronic conditions and were discharged from the intermediate care unit. Twenty-five adults agreed to participate and completed the baseline survey. The change in symptom burden and healthcare utilization at 3 months follow-up compared to baseline was examined using the Wilcoxon Signed-Rank test. The participants’ mean (± SD) age was 67 ± 7 years; 52% were women, 44% had some college/associated degree; 92% were retired/unemployed, and 72% were Black. For the goals discussion component of the program, 83% of the measurable goals were fulfilled. A preliminary analysis of the symptom burden found that the level of energy significantly improved at the 3-month follow-up (p =.032). Significant decreases in hospitalization (p =.01) and critical care admission (p <.01) were found at 3 months post-discharge compared to 3 months before program initiation. In summary, the COORDINATE study could be a pivotal initiative in addressing the holistic care needs of older adults living with multimorbidity. This intervention demonstrates promising outcomes, signaling the need for a larger effectiveness trial.

